# THE ROLE OF PAID CAREGIVERS IN HOME-BASED DEMENTIA CARE: FAMILY, PAID CAREGIVER, AND GERIATRICIAN PERSPECTIVES

**DOI:** 10.1093/geroni/igac059.3122

**Published:** 2022-12-20

**Authors:** Deborah Watman, Jennifer Reckrey

**Affiliations:** Icahn School of Medicine at Mount Sinai, New York City, New York, United States; Icahn School of Medicine at Mount Sinai, New York City, New York, United States

## Abstract

Paid caregivers (e.g., personal care aides, home health aides, other direct care workers) provide essential care that allows people with dementia to remain living at home, yet little is known about the lived experience of this care. This project uses multiple perspective, qualitative longitudinal interviews to explore paid caregiver role in home-based dementia care. We conducted one-on-one interviews via telephone or zoom with the family caregiver, paid caregiver, and geriatrician of an individual person with moderate or severe dementia (n=9) living at home in New York City. After an initial interview, up to 2 additional interviews (at 3 and 6 month intervals) were also conducted for a total of 75 interviews with 29 unique respondents. Interviews were audio-recorded, transcribed, and analyzed using the framework method of analysis. Interviews revealed nuanced care arrangements, but paid caregiver role in care remained largely stable over time. Key findings include: (1) Family caregivers played a primary role in determining overall paid caregiver role in care, (2) Paid caregivers describe the emotional components of caregiving (e.g. being “like family”, having patience) more frequently than families or doctors, and (3) Doctors rarely engage with paid caregivers unless family involvement is limited. The unique structure of each triad emphasizes the importance of person-centered dementia home care. Formal care plans may not reflect the nuances of care arrangements and responsibilities. Rather than prescriptive standards for home care, improved communication and clear expectation setting may help meet the complex needs of people with dementia and their families.

